# Exploring the role of obesity and overweight in predicting postoperative outcome of abdominal surgery in a sub-Saharan African setting: a prospective cohort study

**DOI:** 10.1186/s13104-018-3853-0

**Published:** 2018-10-19

**Authors:** Benjamin Momo Kadia, Alain Chichom-Mefire, Gregory Edie Halle-Ekane

**Affiliations:** 1 Grace Community Health and Development Association, Kumba, Southwest Region Cameroon; 2 Faculty of Infectious and Tropical Diseases, London School of Hygiene and Tropical Medicine, London, UK; 3 Faculty of Health Sciences, University of Buea, Buea, Southwest Region Cameroon

**Keywords:** BMI, Obesity, Overweight, Postoperative outcome, Abdominal surgery, Sub-Saharan Africa

## Abstract

**Objective:**

Current literature on the role of excess weight in predicting surgical outcome is controversial. In sub-Saharan Africa, there is extreme paucity of data regarding this issue in spite of the increasing rates of obesity and overweight in the region. This prospective cohort study, carried out over a period of 4 months at Limbe Regional Hospital in the Southwest region of Cameroon, assessed 30-day postoperative outcome of abdominal surgery among consecutive adults with body mass index (BMI) ≥ 25 kg/m^2^. Adverse postoperative events were reported as per Clavien–Dindo classification.

**Results:**

A total of 103 patients were enrolled. Of these, 68.9% were female. The mean age was 38.2 ± 13.7 years. Sixty-four (62.1%) of the patients were overweight and the mean BMI was 29.2 ±4.3 kg/m^2^. The physical status scores of the patients were either I or II. Appendectomy, myomectomy and hernia repair were the most performed procedures. The overall complication rate was 13/103 (12.6%), with 61.5% being Clavien–Dindo grades II or higher. From the lowest to the highest BMI category, there was a significant increase in the proportion of patients with complications; 25–29.9 kg/m^2^: 6.25%, 30–34.9 kg/m^2^: 18.75%, 35–39.9 kg/m^2^: 25.0%, and ≥ 40 kg/m^2^: 66.70%; p = 0.0086.

## Introduction

Obesity and overweight are a global public health problem [1–3]. A rapid rise in the prevalence of obesity and overweight has been observed in both developed and developing countries [1, 3, 4]. Although a lower prevalence is generally reported in African countries, the trend has been towards a rapid rise in the number of obese and overweight people [2]. In Cameroon, the prevalence of obesity in urban areas has been estimated to range between 5.4 to 6.5% in males and 17.1 to 19.5% in females and the trend is also on the rise [5, 6].

From a surgical perspective, the increasing rate of obesity and overweight has been associated with a rise in the number of persons with excess weight who need to undergo surgery [3, 7, 8]. In Western countries, studies have indicated that excess weight is an independent predictor of adverse surgical outcome, with morbid obesity being associated with a higher risk of postoperative death [9–13]. This association is confirmed by some of the rare available reports in Africa [8, 14–17]. With the introduction of laparoscopic surgery, surgical interventions appear to have become much safer in persons with excess weight [18–20]. Notwithstanding the above mentioned, some authors have reported contrary findings on the role of obesity and overweight in predicting surgical morbidity and mortality. Consequently, literature on the association between obesity and overweight and surgical outcome has remained controversial [7, 21–24].

In Cameroon, there has been a gradual improvement in surgical infrastructure especially with the introduction of laparoscopic surgery which is, however, unavailable at regional and district levels of healthcare where over 70% of the population is treated [25]. This setting, where mainly open abdominal surgery is performed, is characterized by extreme paucity of data on postoperative outcome among obese and overweight persons. This study sought to grade adverse postoperative outcome of open abdominal surgery among obese and overweight persons and to assess the variation of postoperative complication rates across body mass index (BMI) strata for obese and overweight persons at a regional hospital in the Southwest region of Cameroon. We hypothesized that obesity and overweight is associated with high complication rates.

## Main text

### Materials and methods

#### Study design and setting

A prospective cohort study was carried out over a 4-month period (November 01st, 2014 to February 28th, 2015) at the surgical and gynaecological wards of Limbe Regional Hospital (LRH). LRH is based in the Southwest region of Cameroon and is a level III referral centre. During the study, the surgical ward had 24 beds and was run by a general surgeon while the obstetrics/gynaecology ward had 24 beds and was managed by 2 obstetricians/gynaecologists. At the time of the study, LRH has 2 functional operative rooms and there was no intensive care unit. Serious cases were referred to Douala where there are larger hospitals with specialized services.

#### Patients

The study population was enrolled by consecutive convenience sampling. We selected overweight and obese patients (BMI ≥ 25 kg/m^2^) aged ≥ 18 years undergoing abdominal surgery, defined arbitrarily as any procedure involving incision and opening of the layers of the abdomen and/or its surroundings for diagnostic or therapeutic purposes, irrespective of whether the peritoneum was opened or not. Repeat surgeries were excluded as well as patients from whom sufficient relevant data could not be obtained.

#### Data sources and measurements

Overweight and obesity was the main exposure variable and was defined by BMI ≥ 25 kg/m^2^. Preoperative BMI was calculated as weight (in kilograms) divided by height (in metres) squared and patients were stratified by BMI into groups defined by the World Health Organization (WHO), with overweight being BMI ≥ 25 kg/m^2^ to < 30 kg/m^2^ and obesity being BMI > 30 kg/m^2^. For elective procedures, weight and height were measured 24 h prior to surgery by the principal investigator (a medical doctor). For patients requiring emergency surgery, BMI was computed from the most recent (at most 3 weeks prior to surgery) weights and heights in their medical records. Other explanatory variables such as demographics, preoperative diagnoses as reported by the attending surgeon or gynaecologist, and American Society of Anaesthesiology (ASA) physical status scores were recorded. ASA scores include: ASA 1: A normal healthy patient; ASA 2: A patient with a mild systemic disease such as treated hypertension or diabetes; ASA 3: A patient with a severe systemic disease that is not life-threatening like poorly treated hypertension or diabetes; ASA 4: A patient with a severe systemic disease that is a constant threat to life such as unstable angina, and recent myocardial infarction or stroke; ASA 5: A moribund patient who is not expected to survive without the operation.

Patients were also followed-up in the operating room where data such as type of anaesthesia, type of procedure and the risk of sepsis (as per the 1999 Centers for Disease Control and Prevention guidelines of prevention of surgical site infection [26]), were recorded. Variables such as age, status of surgery (major versus minor), anaemia (defined as per WHO standards as haemoglobin level of < 13 g/dl in men and < 12 g/dl in women) and type of surgery (elective versus emergency) and surgical wound class whose subcategories could have differential postoperative outcome were considered as potential effect modifiers. Potential confounding variables such as physical status score, and duration of surgery were also noted. The primary outcome was 30-day complications as defined by the Clavien–Dindo classification which is a widely used scoring system for adverse surgical events [3, 27–29]. Based on this classification, there are 5 grades of complications:

- Grade I: no pharmacological, surgical, endoscopic or radiological interventions are required. Allowed therapeutic regimens are drugs such as antiemetics, antipyretics, analgesics, diuretics, electrolytes and physiotherapy. This grade also includes wound infections opened at the bedside.
- Grade II: pharmacological treatment with drugs other than those allowed for grade I complications is required. Blood transfusion and total parenteral nutrition are also included here.
- Grade III: require surgical, endoscopic or radiological intervention.
- Grade IV: life-threatening (including central venous system complications) and requires intensive care.
- Grade V: death of the patient [27].

Complications were diagnosed and reported in patients’ medical records by the attending surgeon and gynaecologist. Secondary outcomes included readmissions, reoperations, and referrals.

#### Data analysis

All data were entered in an MS excel spread sheet and analyzed using Epi-Info version 7 statistical software. Categorical (binary) variables were compared using the Chi square or Fisher’s exact test as appropriate. Bivariate analysis enabled the determination of other factors (confounders and effect modifiers) that were potentially associated with postoperative complications in our cohort. Means and proportions were compared using Student’s two-sample t-tests (when comparing two groups) or ANOVA tests (when comparing more than 2 groups). The threshold for statistical significance was set at p < 0.05.

### Results

#### Inclusion rate

One hundred and twenty-three persons were potentially eligible for this study. They were all examined for eligibility. There were 116 patients who were confirmed eligible for the study but 13 were excluded because their data were incomplete. Thus, the inclusion rate was 88.8%.

#### Demographic and preoperative characteristics

**Fig. 1:**
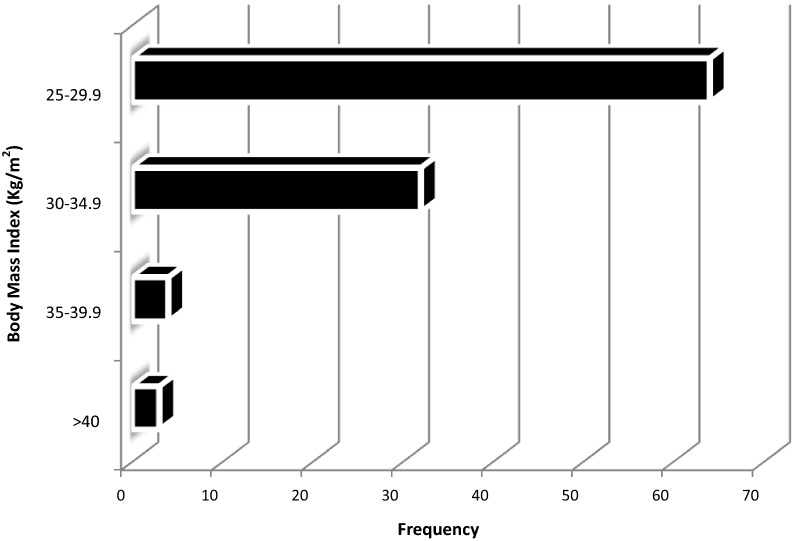
BMI stratification of study population

A total of 103 patients were finally enrolled. There were 32 males, giving a male to female ratio of 0.43. The age range was 18 to 83 years with a mean of 38.2 ± 13.7 years. BMI ranged from 25.0 to 52.1 kg/m^2^, with a mean of 29.2 ±4.3. As shown in Fig. 1, 64 (62.1%) of the patients were overweight and among the 39 obese patients, 7.7% were morbidly obese. Physical status scores were either I or II. Preoperative anaemia was observed in 40 (38.8%) of the patients (Table 1).

**Table 1:** Preoperative and operative characteristics of study population

Variable	n = 103 (%)
Preoperative anaemia	
No	63 (61.2%)
Yes	40 (38.8%)
Status of surgical procedure	
Elective	52 (50.5%)
Emergency	51 (49.5%)
Type of surgical procedure	
Major	59 (57.3%)
Minor	44 (43.7%)
Type of anaesthesia	
General	77 (74.8%)
Locoregional	16 (15.5%)
Regional	10 (9.7%)
Estimated blood loss (ml)	
< 1000	94 (91.3%)
> 1000	9 (8.7%)
Surgical wound class	
Non septic	70 (68%)
Septic	33 (32%)
Perioperative transfusion	
No	86 (83.5%)
Yes	17 (16.5%)
Duration of surgical procedure (h)	
≤ 2	82 (79.6%)
> 2	21 (20.4%)

#### Operative characteristics

A total of 51 (49.5%) of surgical procedures were performed as emergencies and 59 (57.9%) were major procedures (Table 1). Major procedures included myomectomy and laparotomy for ectopic pregnancy. Minor procedure included hernia repair and appendectomy. The most frequently performed surgical procedures were appendectomy, hernia repair and myomectomy. Of the 103 patients, 73.79% were operated on under general anaesthesia and 16.5% patients required perioperative blood transfusion.

#### Postoperative outcome

A total of 13 complications were recorded (12.6% complication rate). Majority of complications (93.9%) were Clavien–Dindo grades 1 and 2 (minor complications) and the rest (6.1%) were grade 3 or higher (major complications). No cases of readmissions, reoperations, deaths or referrals were registered. Differential analysis of complication rates indicated that from the lowest to the highest BMI strata, there was a significant increase in proportion of patients who developed postoperative complications (Table 2). Bivariate analysis of potential confounders and effect modifiers including gender (male versus female), age (> 38 years or ≤ 38 years), comorbidities like diabetes and hypertension, duration of procedure (≤ 2 h or > 2 h), surgical wound class (non-septic versus septic), status of procedure (major versus minor), preoperative anaemia, estimated blood loss (< 1000 ml or ≥ 1000 ml) and type of anaesthesia (general versus regional), was remarkable in that the occurrence of complications was significantly higher among patients of age > 38 years compared to patients of age ≤ 38 years (21% versus 5.4%, p = 0.016).

**Table 2:** Variation of complication rates across BMI strata

BMI (kg/m^2^)	Complications	No complication	Complication rate (%)	p value
25–29.9 (n = 64)	4	60	6.25	0.0086
30–34.9 (n = 32)	6	26	18.75	
35–39.9 (n = 4)	1	3	25	
≥ 40 (n = 3)	2	1	66.7	
Total (103)	13	90	12.62	

### Discussion

This prospective study intended to grade postoperative complications of abdominal surgery among overweight and obese persons and to determine if there is an association between BMI strata and complications. Based on the results of the study, obese and overweight patients undergoing abdominal surgery had good Physical status scores and anaemia was a frequent associated condition. The overall complication rate was rather high and the relative risk seemed to increase with BMI. Furthermore, when complications occurred, they tended to be of low severity.

African countries are characterized by an alarming lack of reports on the epidemiology and morbidity of obesity and overweight and its possible bearing on the management of surgical patients. This study is one of the rare focusing on the outcome of abdominal surgery in obese and overweight patients in sub-Saharan Africa. To the best of our knowledge, it is the first documented Cameroonian study primarily designed to investigate whether obesity and overweight is associated with higher risk of postoperative complications. It has the merits of comparing postoperative outcome across BMI strata and the prospective nature of the study as well as the use of a validated method of reporting surgical outcome increase the level of evidence proposed and external validity of the results. Our findings contribute to addressing current controversies on the impact of obesity on surgical outcomes and attempt to contribute to solving the crucial problem of the alarming lack of data and guidelines for surgeons practicing in sub-Saharan African countries.

The mean BMI in our cohort was low in comparison to many studies assessing postoperative outcome among patients with excess weight because these studies assessed obese patients as a single group [13, 24, 30–32]. It is frequently reported that obese patients undergoing surgery have a high prevalence of comorbidities such as diabetes, hypertension and sleep apnea which can adversely affect surgical outcome [24]. Our patients were generally overweight which is associated with a lower risk of comorbidities. A low rate of comorbidities could explain the overall good physical status score and the low severity of complications observed. According to current literature, the relation between excess weight and surgical outcome is controversial. Obesity has long been pointed as an independent risk factor of postoperative morbidity and mortality [9, 11, 13, 24, 30, 31, 33–35]. The relative risk is described as BMI dependent [9, 13, 15, 36, 37] as observed in our study. Specific complications have been identified at all the steps of the surgical management process. In the pre-operative phase, they seem to be mostly related to the respiratory problems often displayed by obese patients which sometimes result in the need to initiate or prolong the stay in the Intensive Unit with ventilator support [13, 24, 31, 38]. Intra-operatively, morbidity is dominated by bleeding and a longer operative time probably due to operative ergonomics and poorer exposure of the surgical site [33, 39]. Post-operative morbidity is dominated by the risk of local sepsis and abdominal wall defect [24, 30, 34, 35, 40]. Other complications which seem to be frequent in obesity such as myocardial infarction and multi-organ failure did not appear in our report, probably because they are more frequent in morbidly obese patients [7, 13, 34].

Obesity-related risks appear to have significantly reduced with the introduction of laparoscopy, especially for procedures popularly known as providers of postoperative complications in the obese [18–20, 41–44]. Nonetheless, the association between obesity and adverse surgical outcome may not be clear-cut as contradictory reports are available [7, 32, 40, 45]. It is often suspected that the numerous previous reports pointing obesity as a risk factor of adverse surgical outcome may have inspired the over-reporting of postoperative complications in obese patients [46]. We limited such bias by reporting complications using a validated (standard) scoring system. Furthermore, obesity and overweight were estimated using a standard method (BMI) and the estimates were done by medical doctors. These reduced the chances of measurement bias regarding the predictor variable of interest (BMI). However, the magnitude of the relative risk of complications across BMI strata may have been increased by confounders and effect modifiers and this may have led to bias away from the null hypothesis.

In conclusion, the findings of this study suggest that among adults with excess weight, increased BMI seems to be associated with adverse postoperative outcome of abdominal surgery. Special preparation and monitoring may therefore be justified for obese and overweight patients in our setting. Obese and morbidly obese persons undergoing abdominal surgical procedures seem to require more caution and consequently, appropriate measures such as prolonged assisted ventilation and aggressive infection prevention plans should be considered when handling the postoperative period.

## Limitations

The global lack of consistency in the definition of obesity and in particular the paucity of data on obesity-related surgical morbidity in developing countries limited the interpretation of our findings. Furthermore, this study used BMI as measure of adiposity albeit it is suggested that the percentage of fat contributing to total body weight and the body distribution of fat are key in understanding health risks (including surgical outcome) associated with excess weight [7, 30]. Although the small sample size as well as lack of a control group and sensitivity analysis limit the external validity of the results, standard methods were used to measure adiposity and report complications which increase the validity of our findings. A larger prospective study possibly including control groups and comparative analyses of different measures of adiposity as predictors of surgical outcome could have better defined the effect of excess weight on postoperative outcome.

## Abbreviations

ASA: American Society of Anaesthesiologists
BMI: body mass index
WHO: World Health Organization
